# A48 OPTIMIZATION OF TRYPTOPHAN-CONTAINING DIETS TO ACTIVATE THE ARYL HYDROCARBON RECEPTOR AND REDUCE SUSCEPTIBILITY TO COLITIS

**DOI:** 10.1093/jcag/gwab049.047

**Published:** 2022-02-21

**Authors:** L Rondeau, D Haas, J Linton, X Wang, A CAMINERO FERNANDEZ

**Affiliations:** McMaster University Faculty of Health Sciences, Hamilton, ON, Canada

## Abstract

**Background:**

The intestinal microbiota, diet, and the immune system have all been proposed to contribute to the development of inflammatory bowel disease (IBD). The aryl hydrocarbon receptor (AhR) is a critical regulator of intestinal immunity and mucosal barrier homeostasis that is activated by agonists such as diet-derived microbial tryptophan (Trp) metabolites. Increasing evidence suggests that IBD patients have reduced AhR agonists in intestinal content resulting in the downregulation of AhR-regulated genes. In mice, impaired Trp metabolism and AhR signaling translate to increased intestinal inflammation and injury during colitis. This finding highlights the utility of Trp dietary intervention to reduce the onset and progression of inflammation and injury in IBD.

**Aims:**

To optimize a Trp-enriched diet formulation to increase AhR activation and reduce intestinal inflammation and injury in mouse models of colitis.

**Methods:**

Three diet types with elevated Trp were explored: (1) free amino acid diet with added Trp, (2) purified protein diet with added Trp, and (3) purified high protein diet. Following three weeks of diet consumption by C57BL/6 mice, mucosal injury was induced with 2% dextran sulfate sodium in drinking water (DSS) before sacrifice. Susceptibility to colitis was assessed by analyzing stool consistency and blood, microscopic damage, immune infiltration by immunohistochemistry, and pro-inflammatory gene expression (NanoString). Activation of AhR was measured in feces using an *in vitro* AhR luciferase reporter assay. Colonic expression of AhR pathway genes *Cyp1a1*, *Il22*, *Ahrr,* and *Il17* was evaluated by RT-qPCR. Fecal microbiota was analyzed by 16S rRNA gene sequencing (Illumina).

**Results:**

AhR activation *in vitro* and colonic AhR pathway gene expression were elevated in mice fed diets containing added Trp (1 & 2) in comparison to mice fed a high protein diet (3). While all DSS-treated mice developed colitis, mice fed a purified protein diet with added Trp (2) were protected from severe colitis and developed less microscopic damage, immune cell infiltration, and pro-inflammatory gene signalling. Enriched Trp concentrations in the form of free amino acid (1) and high protein (3) diets were not associated with protection from inflammation and injury during colitis.

**Conclusions:**

Our findings suggest that the addition of Trp to a conventional diet (2) may increase microbial tryptophan metabolites to ameliorate colitis severity and intestinal homeostasis through AhR activation. Despite having similar Trp concentrations, a Trp-enriched free amino acid diet (1) and a high protein diet (3) do not improve colitis severity. Thus, both the diet formulation and the availability of Trp should be considered when designing dietary interventions for the treatment of colitis through AhR activation.

**Funding Agencies:**

CCC, CIHRFarncombe Family Digestive Health Research Institute, Douglas Family Chair in Gastroenterology Research, Biocodex Microbiota Foundation

